# Complex Presentation of Congenital Heart Block and Coexisting Congenital Heart Disease in a One-Year-Old Girl: A Case Report

**DOI:** 10.7759/cureus.60720

**Published:** 2024-05-20

**Authors:** Anirudh Kommareddy, Vaibhav Raut, Keta Vagha, Chaitanya Kumar Javvaji, Ashish Varma, Jayant D Vagha, Shailesh Wandile, Ajinkya Wazurkar

**Affiliations:** 1 Pediatrics, Jawaharlal Nehru Medical College, Datta Meghe Institute of Higher Education and Research, Wardha, IND; 2 Cardiology, Jawaharlal Nehru Medical College, Datta Meghe Institute of Higher Education and Research, Wardha, IND

**Keywords:** systemic lupus erythromatosus, cardiac pacemaker, pda (patent ductus arteriosus), ventricular septal defect (vsd), congenital complete heart block

## Abstract

Congenital complete heart block (CCHB) is a rare and potentially life-threatening condition, often associated with maternal autoantibodies. We present the case of a one-year-old girl with recurrent respiratory symptoms, ultimately diagnosed with CCHB and congenital heart disease. She exhibited bradycardia and signs of congestive heart failure. A diagnostic workup revealed significant cardiac abnormalities, including dilated chambers, ventricular septal defect, and patent ductus arteriosus. Serological tests for maternal autoantibodies were negative. The child’s parents opted for discharge without surgical intervention. This case underscores the importance of comprehensive evaluation and management strategies in patients with congenital heart block, particularly in resource-limited settings.

## Introduction

A congenital complete heart block (CCHB) is an uncommon, life-threatening, and permanent condition found in approximately one out of every 20,000 live-born infants. It is often linked to maternal autoantibodies and can be identified in gestation, during infancy, or early childhood. Rarely is it linked to a congenital structural cardiac abnormality [1]. A CCHB is an entire atrial to ventricular impulse blockage. Poor cardiac output, fetal hydrops, and even mortality may arise from the ventricle's independent beating in a ventricular or junctional rhythm, typically occurring at 40-80 beats per minute [2]. Significant cardiac structural anomalies, particularly atrioventricular septal defects and left atrial isomerism, are linked to approximately 50% of fetal cases with CCHB [3]. In children with normal cardiac architecture, there is a significant association between entire congenital heart blocks and maternal connective tissue illness when the mother has positive anti-Sjogren's-syndrome (SS)-related antigen SSA/Ro or SSB/La autoantibodies. Before they reach adulthood, between 63% and 89% of patients acquire a permanent pacemaker; most have them installed within a month of the patient's birth [4]. We present a unique and challenging case of a CCHB patient, age one, who had congenital heart disease. This case highlights the importance of a comprehensive approach while searching for congenital heart block.

## Case presentation

**Figure 1:**
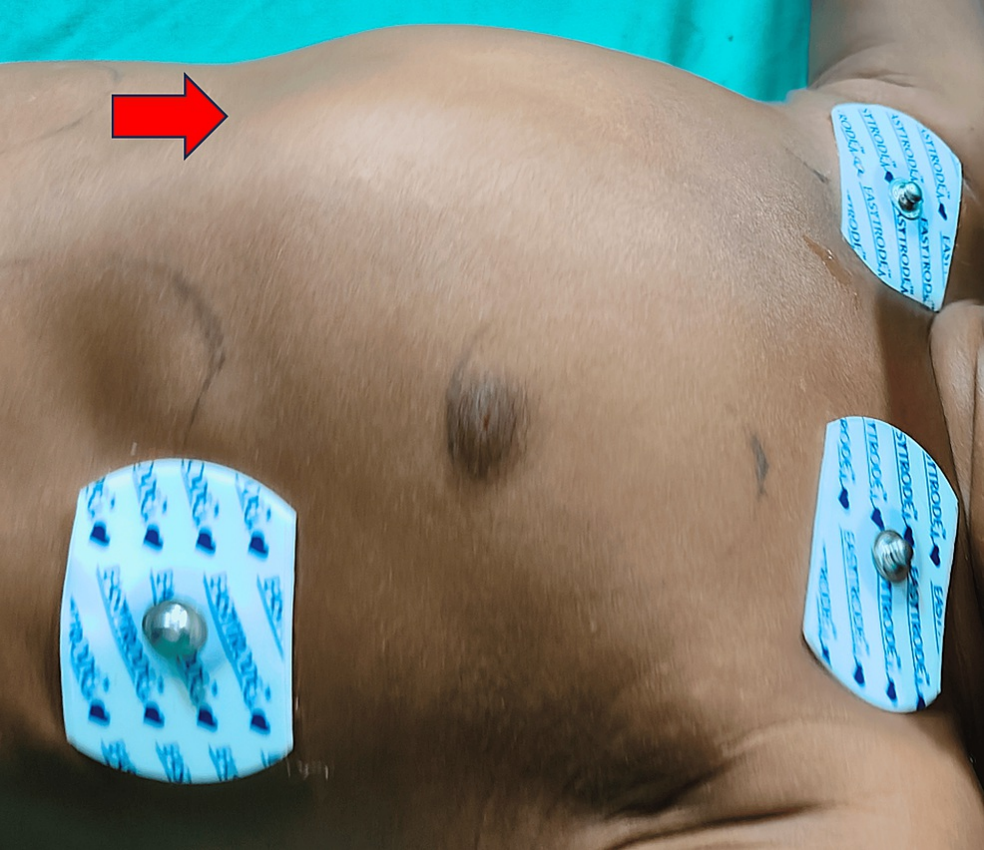
A cardiovascular system examination showing a precordial bulge (red arrow)

A one-year-old girl presented with a history of frequent colds, cough, and fever of over six months. According to the father, she was previously well until the onset of respiratory symptoms, prompting treatment at a local hospital. However, her condition failed to improve, leading to her transfer to our facility with suspected congenital heart disease. History of suck rest cycle and forehead sweating was present. The antenatal history was uneventful. The child was admitted to the pediatric ward. On admission, vital signs revealed bradycardia, with a heart rate of 48 beats per minute and a respiratory rate of 26 breaths per minute. Pulse oximetry indicated oxygen saturation levels of 98% in the right arm and 88% in the right leg. A cardiovascular examination revealed a precordial bulge (Figure 1). On auscultation pan systolic murmur was heard. Other system examinations were normal.

**Figure 2:**
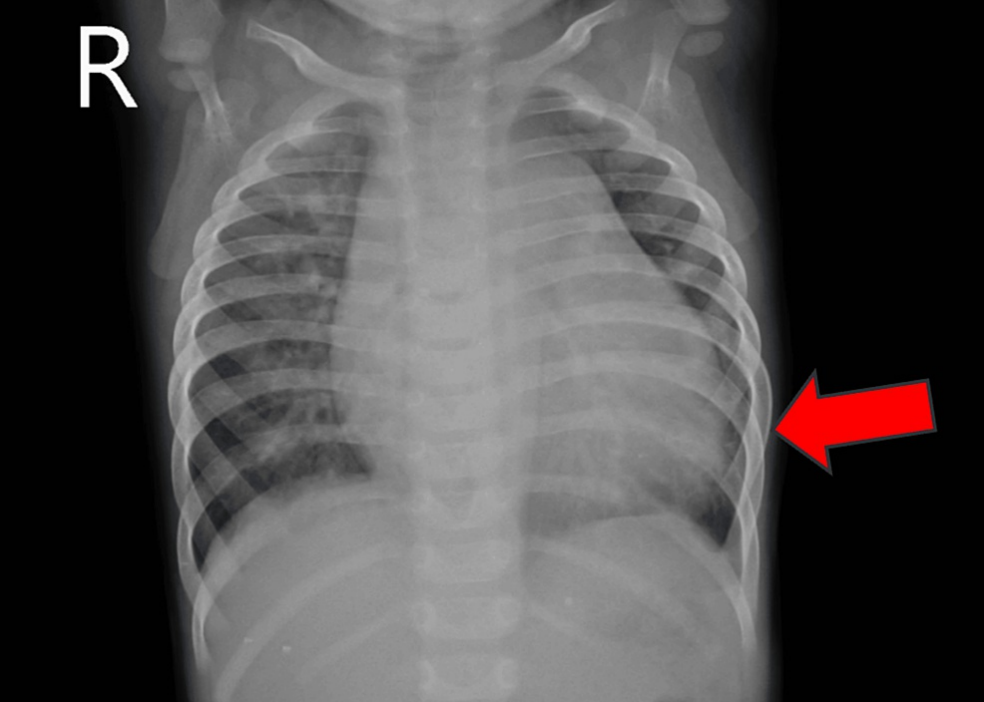
A chest X-ray showing cardiomegaly (red arrow)

Chest X-ray findings suggested cardiomegaly (Figure 2).

**Figure 3:**
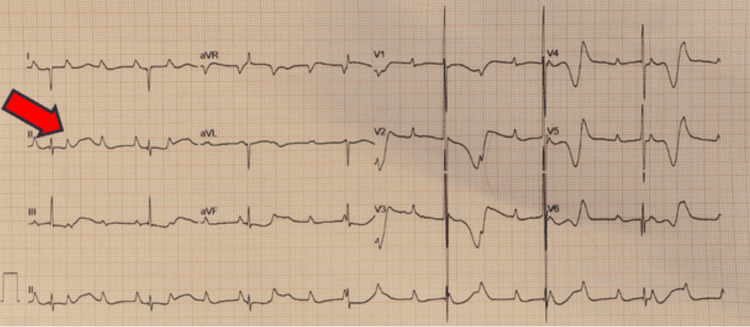
Electrocardiography demonstrated atrioventricular disassociation with marching P waves, complete absence of correlation between P waves and QRS complexes, and narrow QRS representing a suprahisian atrioventricular complete heart block (red arrow)

Electrocardiography (ECG) demonstrated atrioventricular disassociation with marching P waves, the complete absence of correlation between P waves and QRS complexes, and narrow QRS representing suprahisian atrioventricular complete heart block (Figure 3).

**Figure 4:**
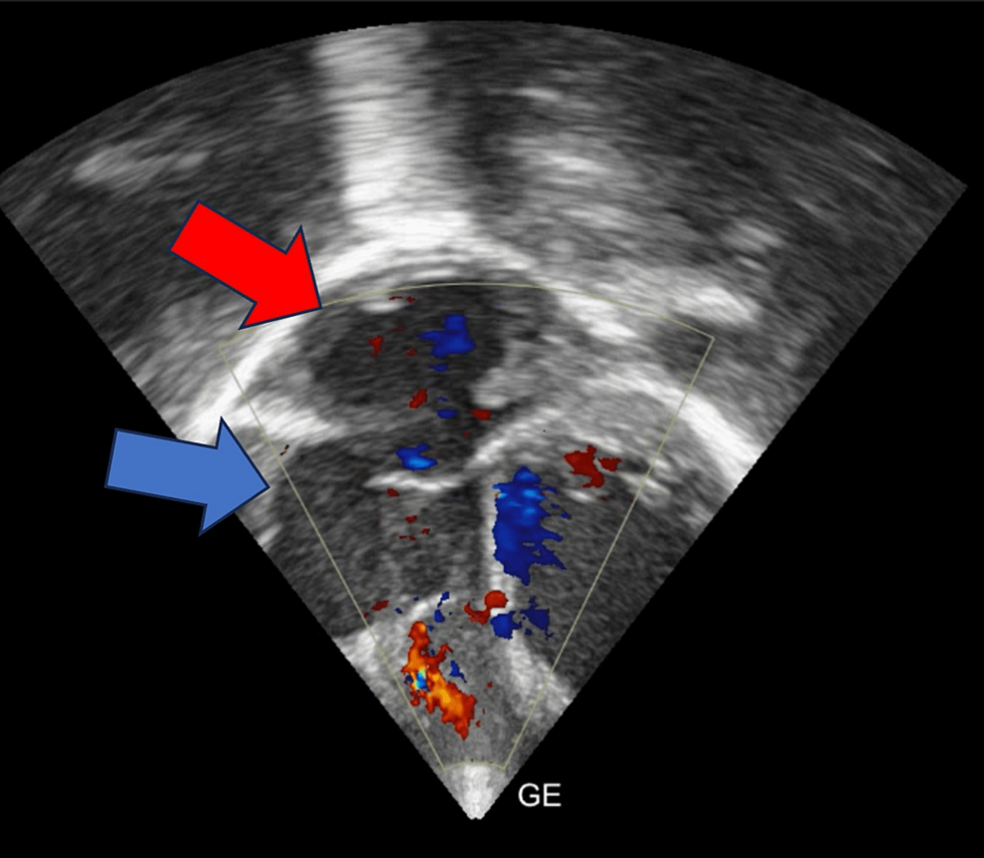
A 2D echocardiogram revealing dilated right atrium (blue arrow) and right ventricle (red arrow)

**Figure 5:**
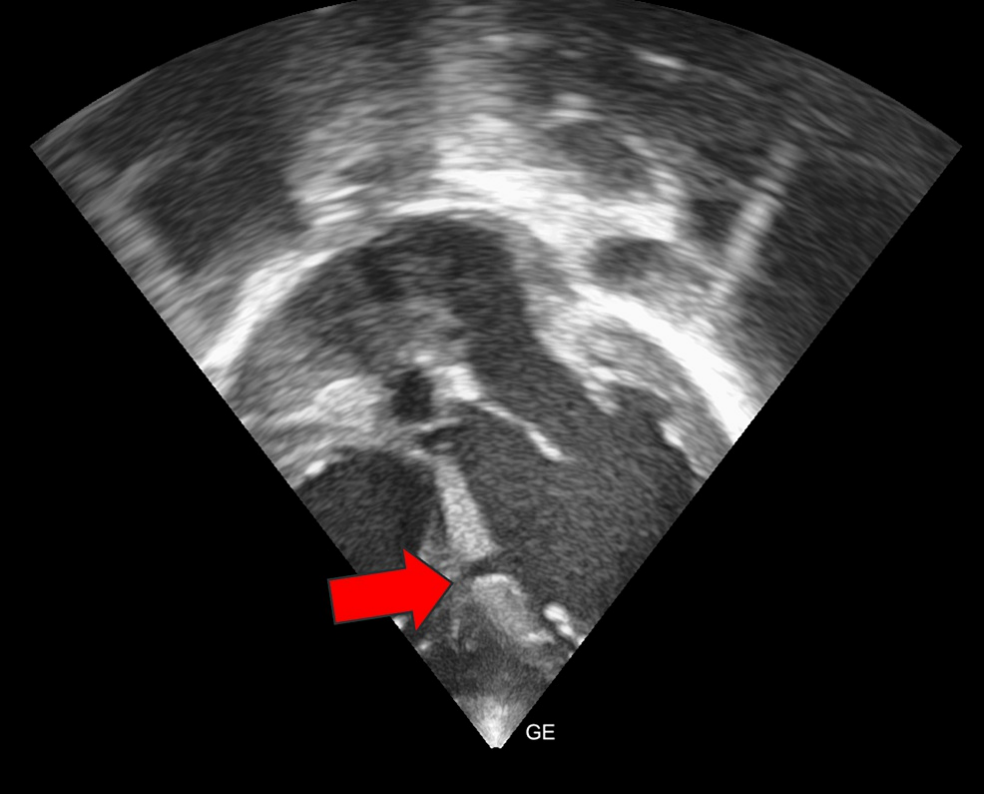
A 2D echocardiogram revealing a ventricular septal defect (red arrow)

**Figure 6:**
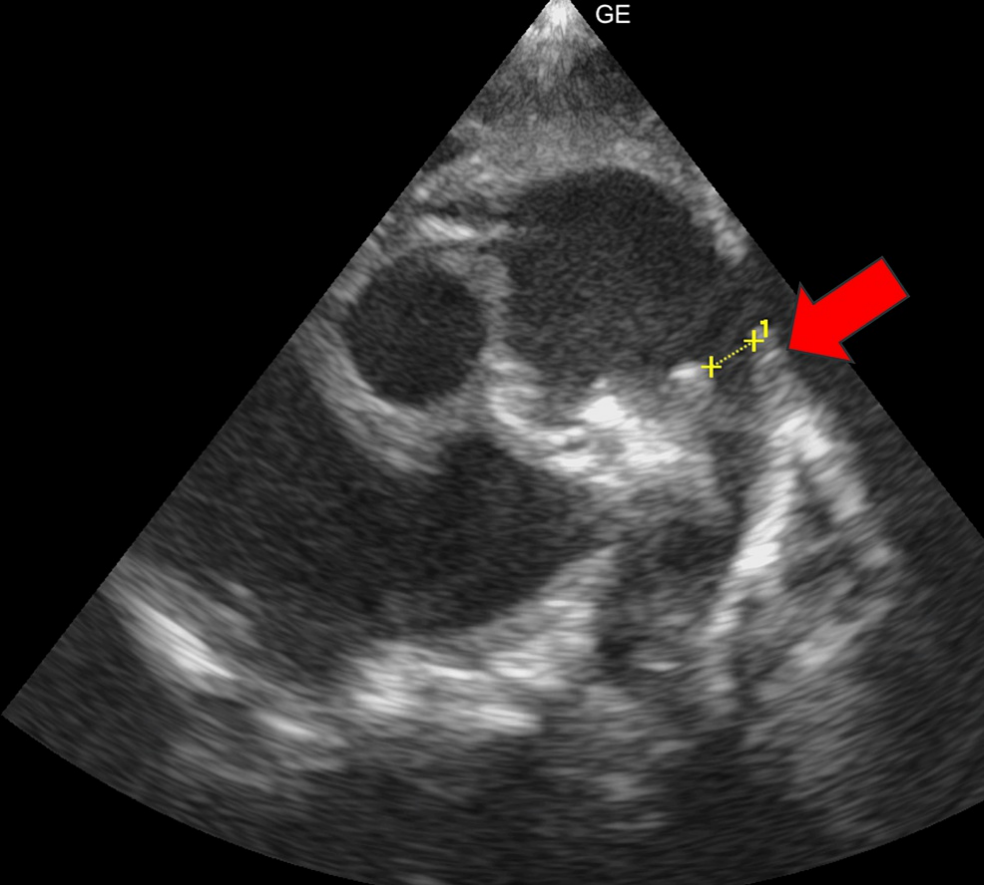
A 2D echocardiogram revealing patent ductus arteriosus (red arrow)

Further investigations revealed an increased total leukocyte count of 15,800/cumm. Other laboratory investigations are normal. The initial management regimen included syrup augmentin, furosemide, tablet orciprenaline, and syrup calcium. Serum anti-Ro and anti-La antibodies were negative, and the mother had no history of systemic lupus erythematosus during pregnancy. A subsequent 2D echocardiogram revealed a dilated right atrium and right ventricle, ventricular septal defect (VSD) with bidirectional shunt, and patent ductus arteriosus (PDA) left to right shunt (Figures 4-6). Pulmonary arterial hypertension and bradycardia were also noted.

**Figure 7:**
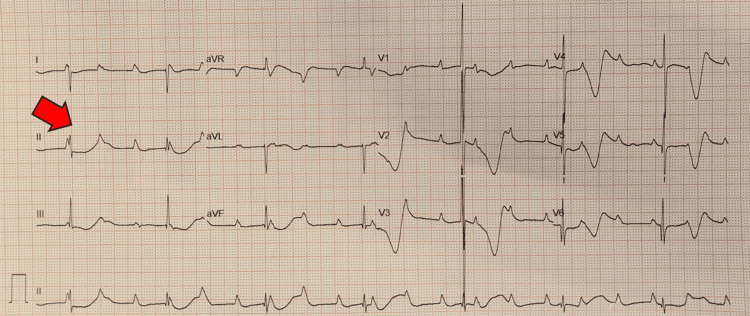
An electrocardiography after atropine test revealing no increase in heart rate or change in electrocardiography, consistent with a high-grade atrioventricular block (red arrow)

An atropine test revealed no increase in heart rate or change in ECG, consistent with a high-grade atrioventricular block (Figure 7).

The patient was deemed to require an epicardial pacemaker, preferably dual-chamber, along with PDA ligation to prevent complications such as heart failure, valvular regurgitation, and exercise-induced syncope. However, the child’s parents expressed unwillingness to proceed with surgical intervention at this time. Hence, the child was discharged against medical advice.

## Discussion

CCHB is a severe condition that, in the past, posed significant risks to affected individuals. However, with the advancements in medical care, the prognosis for this condition has improved dramatically, particularly in developed countries. In these regions, prenatal screening methods, such as fetal echocardiography, enable the early detection of heart block while the baby is still in the womb [5]. Unfortunately, in resource-limited settings where access to advanced medical technology is limited, the prenatal diagnosis of congenital heart block is rare. As a result, the condition is typically identified only after birth or during infancy. This delay in diagnosis can present challenges for timely intervention and management, underscoring the importance of improving healthcare infrastructure and access to prenatal screening in these regions [5].

The literature indicates that CCHB affects an estimated 15,000-20,000 live births. In some instances, a congenital heart block may occur in conjunction with complex structural heart diseases, such as heterotaxy syndrome, which can significantly increase the complexity and severity of the condition. In cases where congenital heart block is associated with heterotaxy syndrome, fetal mortality rates remain high, even with the implementation of pacing interventions. Furthermore, early fetal development exposure to maternal Ro/La autoantibodies may be the secondary cause of congenital heart block. In these cases, affected infants typically require pacemaker placement early in life because of the severity of the block. However, despite interventions, individuals with congenital heart block associated with maternal autoantibodies may still face a higher mortality rate compared to other forms of congenital heart block. This underscores the importance of early detection, appropriate management, and ongoing monitoring for infants at risk of congenital heart block [6].

Defining the precise incidence of CCHB in the general population poses challenges because of its relative rarity compared to the acquired heart block in adults. Histological studies have attempted to shed light on the localization and etiology of congenital heart block. Postmortem examinations have revealed the presence of aberrant fibrous tissue, either at the level of the atrioventricular (AV) node or the AV bundle, histologically disrupting normal conduction pathways. This interruption results in discontinuity between the atria and AV node or between the AV node and ventricular conducting tissues. His bundle studies have been instrumental in localizing the site of the block, particularly in cases with no accompanying cardiac lesions, with the block most frequently situated proximal to the bundle of His [7]. 

Intrauterine fetal death (IUFD), hydrops fetalis, and poor cardiac output are among the problems that can result from CCHB. According to the literature, IUFD rates range from 9% to 45%, with a clear correlation between them and hydrops fetalis. Patients with CCHB may have reduced cardiac output or compromised cardiac function. In high-risk patients, early temporary pacing eases the transition from deep bradycardia and even asystole to permanent pacemaker insertion. Treatment options that have been described include the intravenous infusion of isoproterenol, atropine, epinephrine, and dopamine [4].

There is a 1-2% chance that babies born to moms who test positive for anti-Sjögren's-syndrome-related antigen SSA/Ro or SSB/La antibodies would grow up with CCHB. These mothers may be asymptomatic, or they may show signs of systemic lupus erythematosus or Sjögren's syndrome. It is well known that fetal cardiac myocytes are the target of maternal immunoglobulin G autoantibodies, which can cause inflammation and conduction system fibrosis. Heart block usually appears between 18 and 24 weeks of gestation in cases of fetal and neonatal lupus erythematosus. The degree of scarring in the conduction system correlates with the severity of the heart block. CCHB or Sjögren's disease may also be linked to specific maternal human leukocyte antigen patterns. As a result of maternal autoimmune autoantibodies, a total AV block is regrettably usually irreversible [8]. 

When maternal autoimmune autoantibodies cause a full AV block, it is usually thought to be irreversible. Still, there is just one known case reported by Escamilla et al. of a mother who was previously undiagnosed with systemic lupus erythematosus, recovering wholly and spontaneously [9]. Within 12 hours after birth, in this rare case, the baby's heartbeat returned on its own to a regular sinus rhythm. Notably, anti-SSA/Ro and SSB/La antibody tests were positive for the mother and child, suggesting an autoimmune etiology. This particular example emphasizes the possibility of unforeseen consequences. It emphasizes the significance of ongoing study and attention to detail in managing congenital heart blocks linked to autoimmune disorders in mothers [9].

Temporary pacing can be approached through various methods, each offering unique advantages and considerations. Transvenous pacing involves the insertion of pacing leads through venous access, which can be internal (e.g., internal jugular, subclavian) or external (e.g., external jugular). Alternatively, epicardial pacing utilizes pacing leads placed on the heart's surface, either through a transcutaneous approach or transesophageal insertion. The choice of approach depends on factors such as patient anatomy, clinical urgency, and available resources, with each method offering specific benefits and potential complications [9].

## Conclusions

In conclusion, the case of this one-year-old girl with CCHB and concurrent congenital heart disease highlights the intricate clinical challenges and diagnostic complexities in pediatric cardiology. Despite the negative maternal autoantibodies, the patient presented with signs of congestive heart block and congenital heart disease, necessitating a comprehensive diagnostic evaluation. This case underscores the importance of multidisciplinary care and tailored management strategies in optimizing outcomes for pediatric patients with complex cardiac conditions, particularly in resource-limited settings, while emphasizing the need for further research to elucidate the optimal treatment approaches and long-term prognoses in similar cases.
